# How Foraging Mode Sculpts Sensory Systems: Morphological Evidence From DiceCT and Histology in Sympatric Lizards

**DOI:** 10.1002/ece3.72042

**Published:** 2025-08-21

**Authors:** Lin Leng, Lei Shi

**Affiliations:** ^1^ Xinjiang Key Laboratory for Ecological Adaptation and Evolution of Extreme Environment Organism, College of Life Sciences Xinjiang Agricultural University Urumqi China

**Keywords:** DiceCT, foraging modes, lizards, olfactory system, sensory adaptation, vomeronasal system

## Abstract

The relationship between foraging modes and sensory system morphology is critical for understanding the ecological and evolutionary adaptations of lizards. This study investigates the nasal olfactory system (NOS) and vomeronasal system (VNS) of four sympatric lizards from the Turpan Basin, China, which exhibit distinct foraging strategies: the active foraging *Eremias roborowskii* (Lacertidae), the sit‐and‐wait foraging *Phrynocephalus axillaris* (Agamidae) and *Tenuidactylus dadunensis* (Gekkonidae), and the seasonally frugivorous *Teratoscincus roborowskii* (Sphaerodactylidae), which adopts active foraging during fruit‐searching. Using diffusible iodine‐based contrast‐enhanced computed tomography (DiceCT) and histological sections, we compared the morphology and histology of their NOS and VNS. The results showed significant differences in the nasal cavity and vomeronasal organ structures: active foraging species (*E. roborowskii* and *T. roborowskii* ) exhibited an enlarged nasal cavity with well‐developed lateral nasal conchae, thicker olfactory epithelium (OE), and higher densities of olfactory receptor cells compared to sit‐and‐wait foraging species. The VNS of active foraging lizards also showed thicker vomeronasal sensory epithelium (VSE) and greater vomeronasal receptor cell densities, particularly in *E. roborowskii*. In contrast, sit‐and‐wait foraging *P. axillaris* displayed reduced nasal conchae, thinner OE and VSE, and fewer receptor cells. Interestingly, the seasonal active foraging *T. roborowskii* demonstrated NOS enhancements akin to obligate active foraging species, suggesting a link between fruit detection and olfactory specialization. These findings support the hypothesis that foraging modes drive morphological divergence in the olfactory systems of lizards, highlighting the role of sensory adaptations in ecological niche specialization. This study provides novel insights into the coevolution of sensory structures and foraging behavior in sympatric lizards. Further studies are needed to explore the functional implications of these morphological differences.

## Graphical abstract

The relationship between foraging modes and sensory system morphology is critical for understanding the ecological and evolutionary adaptations of lizards. This study investigates the nasal olfactory system (NOS) and vomeronasal system (VNS) of four sympatric lizards from the Turpan Basin, China, which exhibit distinct foraging strategies. Using diffusible iodine‐based contrast‐enhanced computed tomography (DiceCT) and histological sections, we compared the morphology and histology of their NOS and VNS. The results support the hypothesis that foraging modes drive morphological divergence in the olfactory systems of lizards, highlighting the role of sensory adaptations in ecological niche specialization. This study provides novel insights into the coevolution of sensory structures and foraging behavior in sympatric lizards.

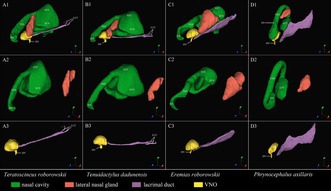

## 1 Introduction

The ability to sense changes in the environment is critical to the survival and reproduction of animals (Verwaijen and Van Damme 2007). Natural selection favors the evolution of sensory systems that show the best function in a given environment (Kimchi and Terkel 2001; Hagelin 2004), but there are also trade‐offs between different sensory organs (Erudaitius et al. 2024). Animal‐specific sensory systems also affect behavioral choices, which may lead to a synergistic evolution between sensory abilities and behavioral strategies (Donihue 2016). Foraging mode plays a key role in the ecological evolution of Squamata (snakes and lizards) (Baeckens, Herrel, et al. 2017). There is a strong link between foraging modes and the development of sensory systems in snakes and lizards (Schwenk 1993b; Cooper 1994, 1997a; Baeckens, Van Damme, et al. 2017). For example, sit‐and‐wait foraging lizards, which usually ambush at a specific site, primarily use visual cues to detect approaching prey (Cooper and van Wyk 1994; Cooper 1995). Active foraging lizards require frequent movement through the different habitats to find concealed and immobile food, typically exhibit higher tongue‐flick rates, have better chemosensory and prey recognition abilities, and are even able to locate prey using only chemical cues (Auffenberg 1984; Cooper 1995, 1997a).

Snakes and lizards are highly dependent on olfactory systems to perceive chemical information from the environment (Mason and Parker 2010). For example, lizards can use olfactory systems for inter‐individual recognition (Mangiacotti et al. 2020; Labra 2023), mate choice (Vilella‐Pacheco et al. 2021; Martin et al. 2024), food searching (He et al. 2018b; Valido and Olesen 2019), predator avoidance (Kabes and Clark 2016; Cornelis et al. 2019; Cliff et al. 2022) and others. Two anatomically separate olfactory systems operate in squamate reptiles (Kaczmarek et al. 2020): the nasal olfactory system (NOS) and the vomeronasal system (VNS) (Grus and Zhang 2006). The nasal cavity and the vomeronasal organ (VNO) constitute the peripheral organs of the NOS and the VNS, respectively (Halpern 1992; Martínez‐Marcos and Halpern 2009). The nasal cavity of squamates exhibits relatively simple morphology. It consists of the tubular vestibulum, Stammteil (main nasal cavity), and outer choanal tube (Kaczmarek et al. 2020, 2021). The NOS primarily perceives small molecules of high volatility that readily diffuse into the air and can travel long distances, whereas the VNS is more sensitive to large molecules of low volatility (Filoramo and Schwenk 2009). VNS of squamate reptiles is mediated by the behavior of tongue‐flick, which actively collects environmental substrates or airborne macromolecular chemicals via the tongue and transports the collected chemicals near the opening of the vomeronasal duct (Døving and Trotier 1998; Filoramo and Schwenk 2009); then chemicals entering the lumen of the vomeronasal organ are sensed by the vomeronasal sensory epithelium (VSE) (Daghfous et al. 2012). Although most squamate reptiles have these dual olfactory systems, the degree of morphological specialization of the olfactory systems varies considerably among taxa (Cooper 1995, 1997b). It has been suggested that the foraging mode may be the main factor driving morphological variation in the olfactory systems of lizards (Cooper and van Wyk 1994; Cooper 1995; Baeckens, Van Damme, et al. 2017). Although entire lizard families can be constrained to a specific foraging mode, there are many exceptions, particularly in Gekkonidae (Cooper 1995; Donihue 2016; Wehsener and Noss 2022), Agamidae (Cooper and van Wyk 1994; Baeckens, Van Damme, et al. 2017), and Lacertidae (Cooper and Whiting 1999; Verwaijen and Van Damme 2007). On the other hand, there are many “intermediate modes” along a continuum between active foraging and sit‐and‐wait foraging that can be described as mixed strategies (Wehsener and Noss 2022), and sex, tail loss, temperature, seasonality, and lunar phase all contribute to variation in foraging mode (Werner et al. 2004). Baeckens, Van Damme, et al. (2017) analyzed literature data from nearly 100 species using phylogenetic comparative methods to examine whether foraging mode and diet influence baseline tongue‐flick rates. Their results demonstrated that active foragers exhibit significantly higher baseline tongue‐flick rates than ambush foragers. This study concluded that foraging mode, not phylogenetic relatedness, drives convergent evolution of similar levels of squamate chemosensory investigation. The significant differences in foraging mode among different lizard species provide a unique opportunity to reveal the relationship between foraging modes and the olfactory systems (Cooper and Habegger 2000). Nevertheless, morphological variation in these systems across different foraging strategies has received limited attention to date.

Four sympatric desert lizard species (*Eremias roborowskii*, *Phrynocephalus axillaris* , *Tenuidactylus dadunensis*, and *Teratoscincus roborowskii* ) inhabit China's Xinjiang Turpan Basin. *E. roborowskii* (Lacertidae) is an omnivorous lizard; *Capparis spinosa* fruits are also one of its important food sources. Direct field observations revealed *E. roborowskii* feeding on *C. spinosa* fruits, and *C. spinosa* seeds were identified in lizard fecal samples. Species in the family Lacertidae often have a well‐developed olfactory system to detect chemical signals in the environment, and their foraging mode is classified as a characteristic active foraging mode (Baeckens, Van Damme, et al. 2017). *P. axillaris* (Agamidae), a sit‐and‐wait foraging forager, is a species within a family generally considered to rely predominantly on visual cues (Baeckens, Van Damme, et al. 2017). *T. dadunensis* (Gekkonidae) is a sit‐and‐wait foraging predator that primarily feeds on insects, as evidenced by long‐term field observations. In contrast, *T. roborowskii* (Sphaerodactylidae) is a seasonally frugivorous species, with *C. spinosa* fruits constituting its main summer diet (Liu et al. 2010; Yang et al. 2021; Gao et al. 2023). Populations of the typically sit‐and‐wait foraging *Platysaurus broadleyi* living near fig trees adopt an active foraging mode during fruiting seasons to eat dropped fruit (Greeff and Whiting 2000; Whiting 2007). As a result, omnivorous *T. roborowskii* adopt an active foraging mode similar to that of *P. broadleyi* when searching the fixed and concealed *C. spinosa* fruits (unpublished data). In addition, *T. roborowskii* showed significant tongue‐flick behavior in the leachate of *C. spinosa* fruits (He et al. 2018a). Among the four desert lizard species, *E. roborowskii*, *T. dadunensis*, and *T. roborowskii* are all endemic to the Turpan Basin in China (Shi et al. 2002).

In recent years, advances in imaging technology have greatly improved the ability to visualize and analyze the anatomical relationships of tiny tissue structures (Gignac and Kley 2018; Blackburn et al. 2024). In a recent study, the coupling of histological sections and DiceCT has been effectively applied in structural studies of the nasal cavity and VNO in lizards. For example, research has been conducted on *Takydromus sexlineatus* (Baeckens, Van Damme, et al. 2017), *Anolis sagrei* (Kaczmarek et al. 2020), *Lepidodactylus lugubris* and *Eublepharis macularius* (Kaczmarek et al. 2021). The integration of histological sections with DiceCT can more intuitively demonstrate the structural relationship between the NOS and VNS in squamate reptiles (Kaczmarek et al. 2021).

Comparing olfactory system morphology among lizards with distinct foraging modes enhances our understanding of the coevolutionary link between sensory structure variation and ecological behavior in squamate reptiles (Verwaijen and Van Damme 2007). Sympatric lizards often exhibit distinct foraging modes that directly influence their behavior, morphology, and performance (Donihue 2016). Here, we compared the fine structure of the NOS and VNS of four sympatric lizards with different foraging modes, using histological sections and DiceCT. We predict that different foraging modes drive morphological variation in the olfactory systems of lizards. Active foraging species (*E. roborowskii* and *T. roborowskii* ) are more dependent on the olfactory system, with thicker sensory epithelium and more olfactory receptor cells. In contrast, sit‐and‐wait foraging species ( *P. axillaris* and *T. dadunensis*) are thought to rely mainly on the visual system; the olfactory system is less developed than that of active foraging species.

## 2 Material and Methods

### 2.1 Study Animals

**FIGURE 1:**
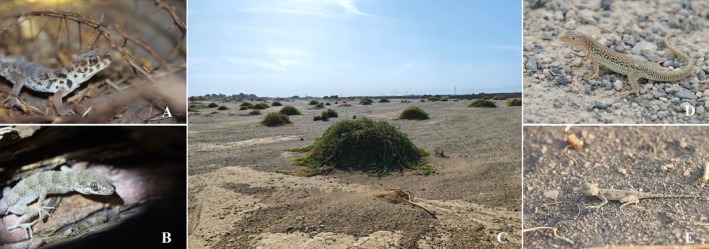
Photos of species and habitats. (A) *Teratoscincus roborowskii* (active foraging); (B) *Tenuidactylus dadunensis* (sit‐and‐wait foraging); (C) habitats; (D) *Eremias roborowskii* (active foraging); (E) *Phrynocephalus axillaris* (sit‐and‐wait foraging).

We captured a total of 17 individuals of lizards, *T. roborowskii* (*n* = 4) (Figure 1A), *T. dadunensis* (*n* = 4) (Figure 1B), *E. roborowskii* (*n* = 4) (Figure 1D) and *P. axillaris* (*n* = 5) (Figure 1E) from one site (Figure 1C) (89°12′35″ E, 42°51′01″ N) in Turpan City, Xinjiang, China, at an altitude of 89 m, on August 2023. All individuals were healthy. These specimens were transported to Xinjiang Agricultural University, where they were housed individually in plastic cages. We measured the snout‐vent length (SVL) of each individual before euthanasia. Measurements were accurate to within 0.01 mm. Adult status was determined based on the minimum SVL of gravid individuals (Zhao et al. 1999; Song 2009; Shi and Zhao 2011; Wang 2012). All sampled individuals had SVLs greater than the minimum observed in gravid specimens, confirming their adult status (Table 1). We euthanized lizards by deep anesthesia with ether followed by rapid decapitation (Yang et al. 2020). After euthanizing a lizard, we removed and preserved the head in 10% buffered formalin (1:20 volume, tissue: solution) for the different research methods (Table 1). The use of animals in this study was approved by the Laboratory Animal Welfare Committee of Xinjiang Agricultural University (Approval No. 2023013). Abbreviated nouns appear in the text (Table S1).

**TABLE 1:** Numbers of lizards used for DiceCT and light microscopy.

Species	Snout‐vent length (SVL)	Minimum SVL of gravid individuals	DiceCT	Light microscopy
*Teratoscincus roborowskii* (active foraging)	SVL = 81.67 ± 2.25 (78.83–84.91) mm	75.52 mm (Song 2009)	*n* = 1	*n* = 3
*Tarentola dadunensis* (sit‐and‐wait foraging)	SVL = 52.31 ± 2.06 (49.13–54.73) mm	33.29 mm (Shi and Zhao 2011)	*n* = 1	*n* = 3
*Eremias roborowskii* (active foraging)	SVL = 65.48 ± 4.16 (58.74–65.38) mm	36.17 mm (Wang 2012)	*n* = 1	*n* = 3
*Phrynocephalus axillaris* (sit‐and‐wait foraging)	SVL = 50.06 ± 2.30 (46.93–52.47) mm	40.00 mm (Zhao et al. 1999)	*n* = 1	*n* = 4

### 2.2 Iodine Staining and DiceCT

One lizard of each species was taken; we first rinsed each head for 2 h in a flowing water rinse to remove the formalin, then immersed in 1.25% (total solute) Lugol's iodine solution (I_2_ + KI + H_2_O), then stained for 14 days in darkness, replaced with a new Lugol's iodine solution every 72 h (Callahan et al. 2021) to enhance the X‐ray contrast of soft tissues (Metscher 2009). The stained head tissues were rinsed with distilled water and gently dried with absorbent paper. Individual head tissues were then wrapped in self‐sealing bags to prevent drying during scanning (Yohe et al. 2018). We secured specimens with fixation clips and scanned them using a ZEISS Xradia 515 Versa 3D X‐ray microscope from the Xinjiang Institute of Ecology and Geography of the Chinese Academy of Sciences for tomography. The results were exported to DICOM format for 3D reconstruction of the NOS and VNS through the Segment Editor in 3D Slicer‐5.7.0 software (Fang et al. 2022; Collin et al. 2024).

### 2.3 Light Microscopy

For histological study, we first rinsed each head for 2 h in a flowing water rinse to remove the formalin. After rinses with running water, the tongues of the four lizard species were photographed with a Canon 90 D. We then decalcified the head to remove the inorganic calcium by placing the specimen in 10% EDTA solution for 15 days; we changed the new EDTA solution daily (Brykczynska et al. 2013). After complete decalcification, each specimen was thoroughly rinsed in continuously flowing tap water for 24 h to ensure complete removal of decalcification agents. To dehydrate tissue prior to embedding, we processed samples through an ethanol (EtOH) series of increasing concentrations as follows: 30% EtOH (2 h), 50% EtOH (2 h), 70% EtOH (2 h), 85% EtOH (2 h), 95% EtOH (1 h), 95% EtOH (2 h), 100% EtOH (2 h), 100% EtOH/xylene (2:1, for 10 min), 100% EtOH/xylene (1:1, for 10 min), 100% EtOH/xylene (1:2, for 10 min), 100% xylene (10 min). We then began infiltration with 100% xylene/paraffin (1:1) solution (56°C, for 10 min), then 100% paraffin (2 h in the oven), 100% paraffin overnight in the oven, all at 58°C. We then embedded each head individually in fresh paraffin in a mold. Serial transverse sections of 10 μm were prepared using a YD‐315 rotary microtome (Jinhua Yidi Medical Equipment Company Limited, China). Paraffin sections were stained using Hematoxylin–Eosin (H.E.) staining (Quinzio and Reiss 2018; Kaczmarek et al. 2020). The NOS and VNS of each lizard were photographed using a Motic BA210 digital microscope (Motic Industrial Group Limited, China). The thickness of the sensory epithelium in the middle of the NOS and VNS of each lizard was measured under 100X magnification using a Motic Images Plus 3.0 micrometry system (Motic Industrial Group Limited, China); each visual field was randomly measured 3 times. The number of olfactory sensory cells and vomeronasal sensory cells in a 60 × 60 μm area in similar parts of each lizard was counted under 400× magnification using the cell counting tool in Adobe Photoshop (24.0.0); one complete cell was counted when half of the cells fell inside the square (Han et al. 2024).

### 2.4 Statistical Analysis

All measurement data were processed with SPSS 25.0. First, the Kolmogorov–Smirnov and Levene's tests were used to assess data normality and homogeneity of variance, respectively. All groups met the assumption of homoscedasticity (*p* > 0.05). Histological parameters of the olfactory epithelium (OE) and the vomeronasal sensory epithelium (VSE) of the four lizard species were compared by one‐way ANOVA, and multiple comparisons were made by the Duncan method. Data are expressed as mean ± standard deviation (mean ± SD), and plots were done in GraphPad Prism 10, with significantly different (*p* < 0.05). Furthermore, we conducted a post hoc power analysis using G*Power 3.1 software to evaluate the statistical power of our one‐way ANOVA comparisons for key histological parameters (Kang 2021). The results indicate that the actual power of our study in detecting differences in the primary parameters of interest exceeded the conventional threshold of 80% for achieving adequate statistical power (Table S2).

## 3 Results

### 3.1 Morphological and Histological Characteristics of the NOS

**FIGURE 2:**
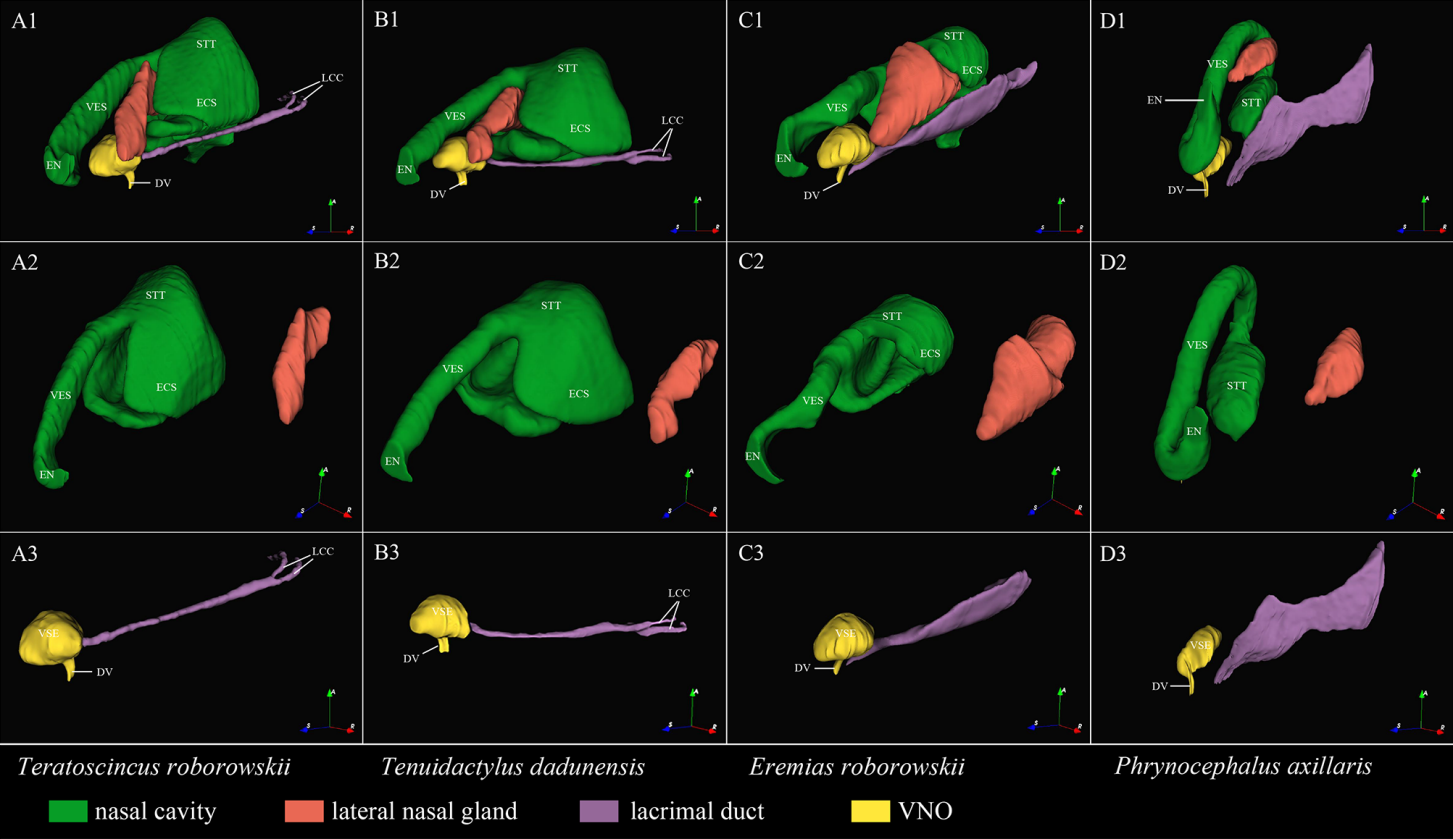
3D reconstruction of nasal cavity and vomeronasal organ of four species of lizards. (A1–A3) *T. roborowskii* (active foraging); (B1–B3) *T. dadunensis* (sit‐and‐wait foraging); (C1–C3) *E. roborowskii* (active foraging); (D1–D3) *P. axillaris* (sit‐and‐wait foraging). DV, VNO duct; ECS, extra‐conchal space; EN, external naris; LCC, lacrimal canaliculi; STT, Stammteil; VES, porch.

**FIGURE 3:**
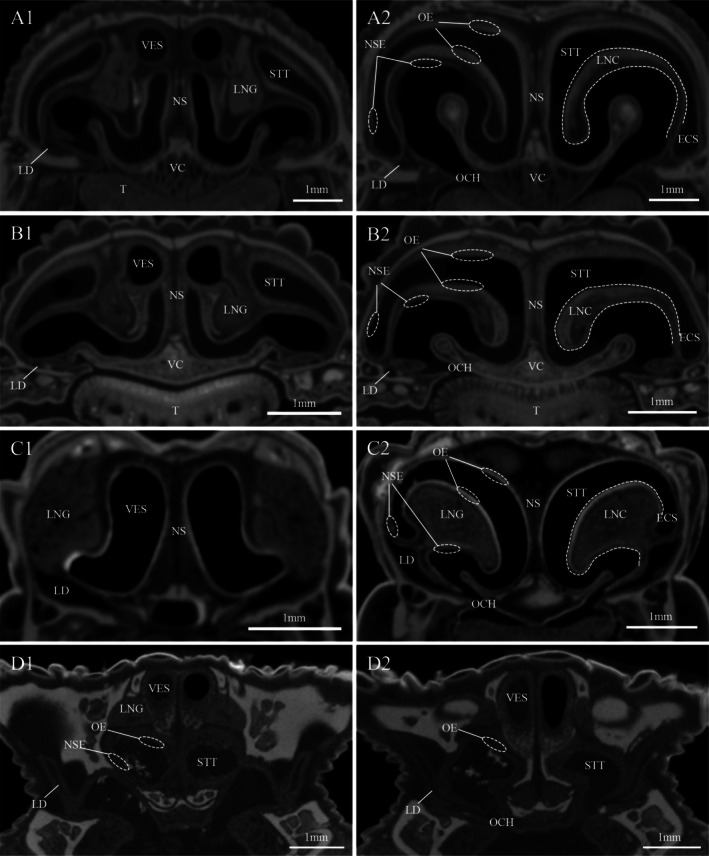
Transverse CT scans of the olfactory system of four species of lizards. (A1, A2) *T. roborowskii* (active foraging); (B1, B2) *T. dadunensis* (sit‐and‐wait foraging); (C1, C2) *E. roborowskii* (active foraging); (D1, D2) *P. axillaris* (sit‐and‐wait foraging). ECS, extra‐conchal space; LD, lacrimal duct; LNC, lateral nasal concha; LNG, lateral nasal gland; NSE, non‐sensory epithelium; OCH, outer choana; OE, olfactory epithelium; STT, Stammteil; T, tongue; VC, vomerine cushion; VES, vestibule.

**FIGURE 4:**
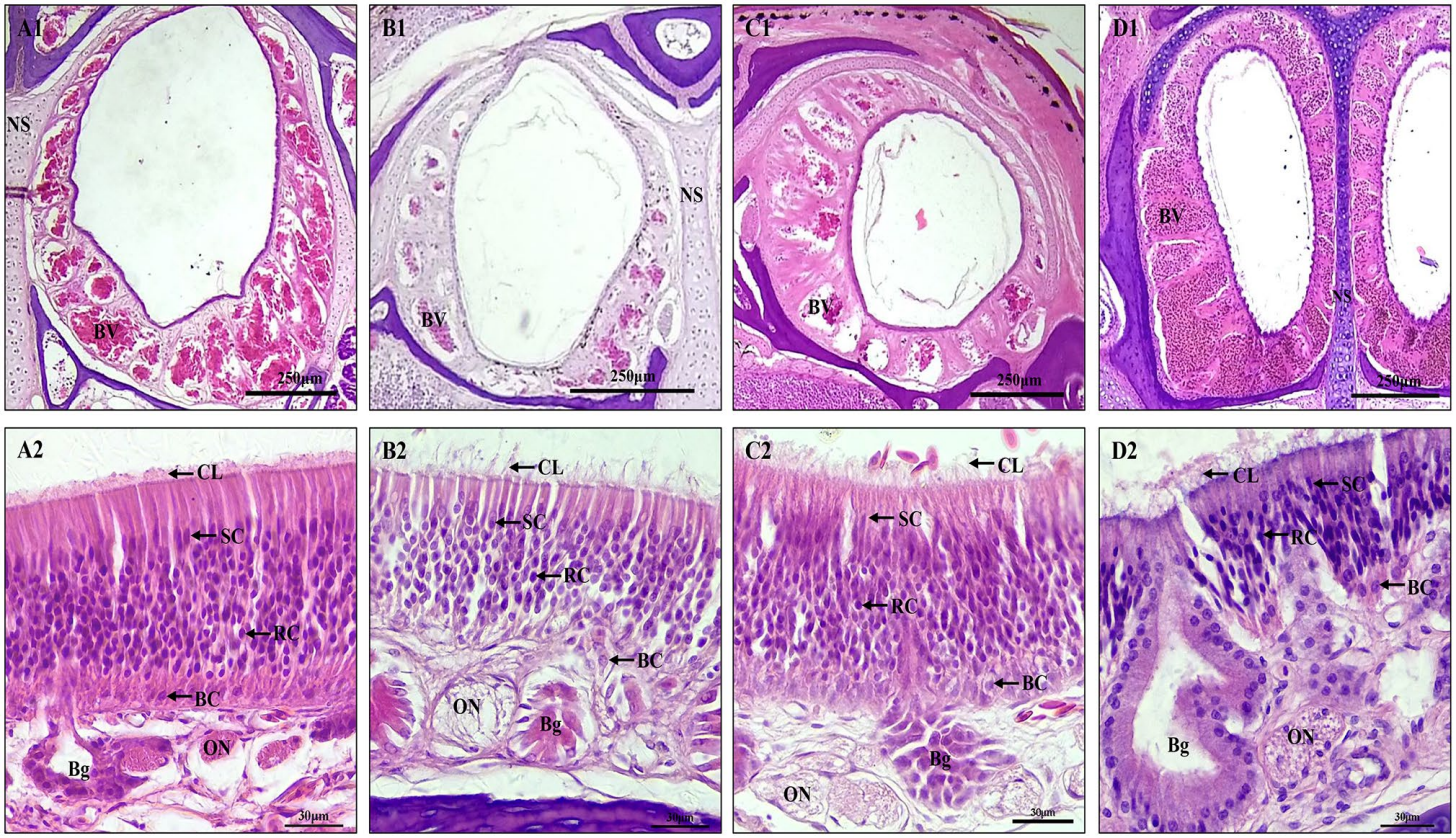
Transverse sections of the vestibulum and olfactory epithelium in four species of lizards. (A1, A2) *T. roborowskii* (active foraging); (B1, B2) *T. dadunensis* (sit‐and‐wait foraging); (C1, C2) *E. roborowskii* (active foraging); (D1, D2) *P. axillaris* (sit‐and‐wait foraging). BC, basal cell; Bg, Bowman's glands; BV, blood vessel; CL, cilia; NS, nasal septum; ON, olfactory nerve; RC, receptor cell; SC, supporting cell.

The results of transverse CT scans and 3D reconstruction of the NOS of the four species of lizards showed a clear morphological differentiation of the nasal cavity (Figures 2 and 3). The nasal cavity of the nocturnal *T. roborowskii* and *T. dadunensis* is similar in morphology; the vestibulum is elongated and tubular, large Stammteil (main nasal cavity), and well‐developed lateral nasal concha forming a large extra‐conchal space (Figure 2A1,A2,B1,B2). The posterior part of *E. roborowskii* of the vestibulum is curved laterally and relatively large, with a well‐developed Stammteil and lateral nasal concha (Figure 2C1,C2). The anterior part of the vestibulum of *P. axillaris* descends almost vertically from the external naris; then it runs posterodorsally and finally bends ventrally to connect with the Stammteil; the Stammteil is small, and no lateral nasal concha were found (Figures 2 and 3). The vestibulum is characteristically lined in four species of lizards, with a stratified keratinized epithelium of variable thickness. It is surrounded by a network of sinusoids‐blood lacunae that lie within a reticulum of smooth muscle fibers. This subepithelial layer is cavernous tissue (Figure 4A1,B1,C1,D1). Lateral nasal glands were present in four species of lizards; the opening of the duct of the lateral nasal gland is at the juncture of the vestibulum and the main nasal cavity (Figure 3A1,B1,C1,D1), with the most developed in *E. roborowskii*, followed by *T. roborowskii* and *T. dadunensis*, and relatively small in *P. axillaris* (Figures 2 and 3).

**FIGURE 5:**
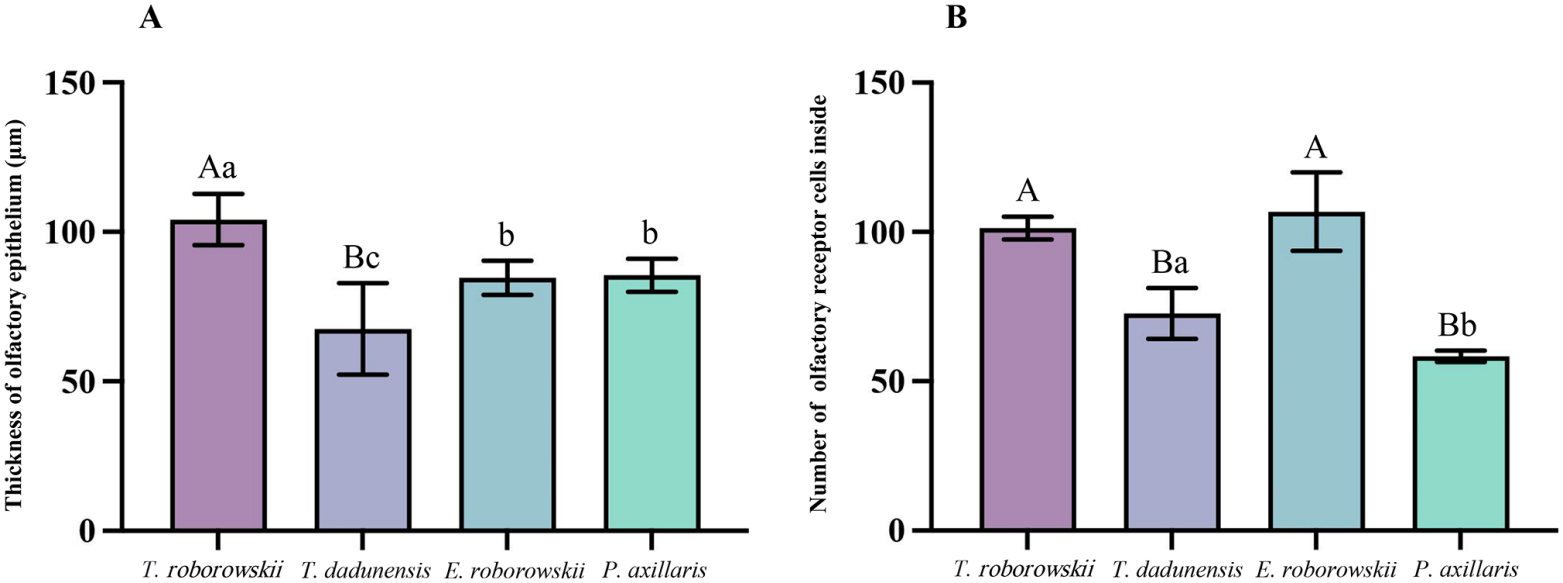
Histological parameter of the olfactory epithelium in four species of lizards. (A) Thickness of the olfactory epithelium; (B) number of olfactory receptor cells within a 60 × 60 μm^2^ area. Groups not sharing the same lowercase letter in each cluster are significantly different (*p* < 0.05). Groups not sharing the same capital letter in each cluster are highly significantly different (*p* < 0.01). *T. roborowskii* (active foraging); *T. dadunensis* (sit‐and‐wait foraging); *E. roborowskii* (active foraging); *P. axillaris* (sit‐and‐wait foraging).

The OE of the four species of lizards was mainly composed of supporting cells, receptor cells, and basal cells (Figure 4A2,B2,C2,D2). Abundant Bowman's glands and olfactory nerves were present at the base of the OE (Figure 4A2,B2,C2,D2). Receptor cells formed the thickest part of the OE; supporting cells were located apically, close to the Stammteil lumen; there were distinct cilia at the outermost periphery of the OE (Figure 4). Highly significant differences were observed in the thickness of the OE of the four species of lizards (*p* = 0.007) (Table 2). The thickness of the OE of *T. roborowskii* was significantly greater than that of the OE of *T. dadunensis* (*p* = 0.001), the OE of *E. roborowskii* (*p* = 0.03), and the OE of *P. axillaris* (*p* = 0.027). The thickness of the OE was significantly greater in *E. roborowskii* than in *T. dadunensis* (*p* = 0.05). The thickness of the OE was significantly greater in *P. axillaris* than in *T. dadunensis* (*p* = 0.032) (Figure 5A). Highly significant differences were noted in the number of olfactory receptor cells of the four lizard species within a 60 × 60 μm^2^ unit area at 400× magnification (*p* < 0.001) (Table 2). The number of olfactory receptor cells in *T. roborowskii* was highly significantly more than that in *T. dadunensis* (*p* = 0.001) and highly significantly more than that in *P. axillaris* (*p* < 0.001). The number of olfactory receptor cells of *E. roborowskii* was highly significantly more than that of *T. dadunensis* (*p* < 0.001) and highly significantly more than that of *P. axillaris* (*p* < 0.001). The number of olfactory receptor cells in *T. dadunensis* was significantly more than that of *P. axillaris* (*p* = 0.033) (Figure 5B).

**TABLE 2:** Foraging mode classification and correlated histological parameters in four lizard species.

Species	Foraging mode	Thickness of the olfactory epithelium (μm)	Number of olfactory receptor cells within a 60 × 60 μm^2^ area	Thickness of the vomeronasal sensory epithelium (μm)	Number of vomeronasal receptor cells within a 60 × 60 μm^2^ area
*Teratoscincus roborowskii*	Active foraging	104.14 ± 8.57 (98.01–113.93) Aa	101.29 ± 3.77 (98.10–105.44) A	172.02 ± 8.20 (162.63–177.81) Aa	69.58 ± 2.11 (67.17–71.08) B
*Tarentola dadunensis*	Sit‐and‐wait foraging	67.53 ± 15.30 (50.41–79.86) Bc	72.71 ± 8.51 (66.75–82.45) Ba	157.67 ± 15.18 (143.95–173.99) a	67.73 ± 1.84 (65.83–67.83) B
*Eremias roborowskii*	Active foraging	84.64 ± 5.74 (80.32–91.15) b	106.47 ± 12.63 (93.58–118.83) A	156.90 ± 32.84 (120.59–184.52) a	97.14 ± 10.50 (86.17–107.08) A
*Phrynocephalus axillaris*	Sit‐and‐wait foraging	85.49 ± 5.49 (81.42–93.50) b	58.35 ± 1.92 (55.83–60.42) Bb	112.41 ± 16.86 (97.35–135.25) Bb	46.28 ± 4.53 (42.78–52.33) C

*Note:* Data are expressed as mean ± standard deviation. Groups not sharing the same lowercase letter in each cluster are significantly different (*p* < 0.05). Groups not sharing the same capital letter in each cluster are highly significantly different (*p* < 0.01).

### 3.2 Morphological and Histological Characteristics of the VNS

**FIGURE 6:**
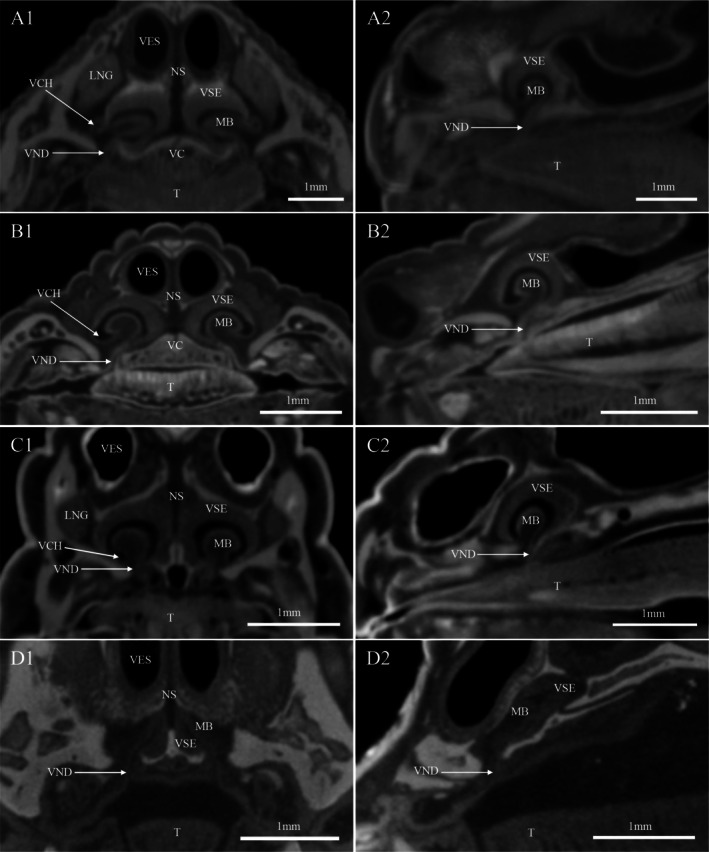
Transverse and sagittal CT scans of the vomeronasal organ of four species of lizards. (A1, A2) *T. roborowskii* (active foraging); (B1, B2) *T. dadunensis* (sit‐and‐wait foraging); (C1, C2) *E. roborowskii* (active foraging); (D1, D2) *P. axillaris* (sit‐and‐wait foraging). LNG, lateral nasal gland; MB, mushroom body; NS, nasal septum; T, tongue; VC, vomerine cushion; VCH, ventral channel; VES, vestibulum; VSE, vomeronasal sensory epithelium; VND, VNO duct.

**FIGURE 7:**
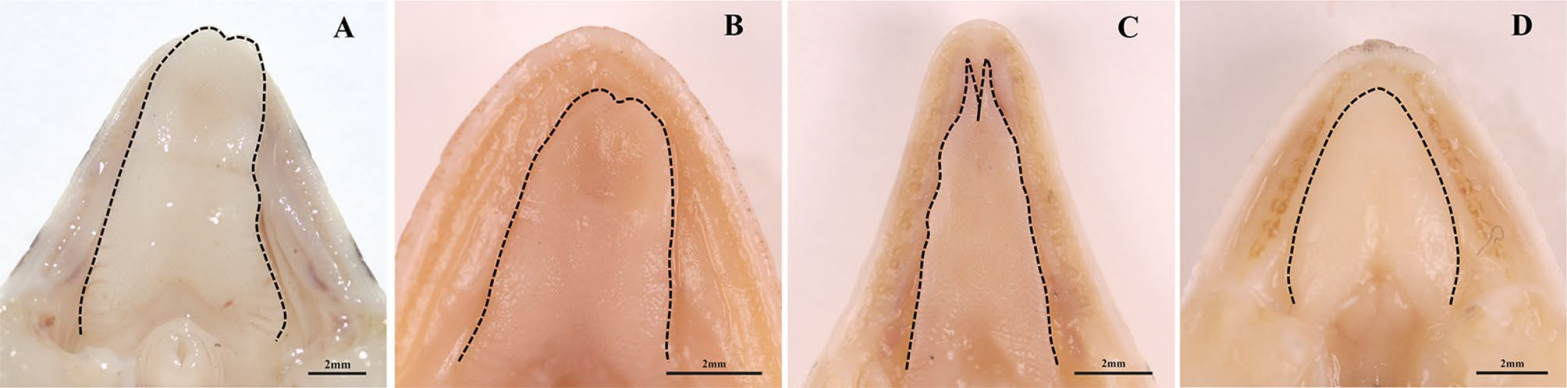
Tongue morphology of four species of lizards. (A) *T. roborowskii* (active foraging); (B) *T. dadunensis* (sit‐and‐wait foraging); (C) *E. roborowskii* (active foraging); (D) *P. axillaris* (sit‐and‐wait foraging).

The paired vomeronasal organ (VNO) of four species of lizards is located below the vestibulum, separated by the nasal septum, and communicates with the oral cavity through the VNO duct (Figure 6A1,B1,C1,D1). Sagittal CT scan results revealed that the VNO of *T. roborowskii* , *T. dadunensis*, and *P. axillaris* was located above the anterior end of the tongue through the opening of the VNO duct (Figure 6A2,B2,D2). The VNO of *E. roborowskii* was located above the bifurcated tongue through the opening of the VNO duct (Figure 6C2). The tongue of *E. roborowskii* was long and distinctly bifurcated (Figure 7C). The tongues of *T. roborowskii* and *T. dadunensis* were long and wide, and there was a slight bifurcation at the tip of the tongue (Figure 7A,B). The tongue of *P. axillaris* was not bifurcated and was broad and short (Figure 7D).

**FIGURE 8:**
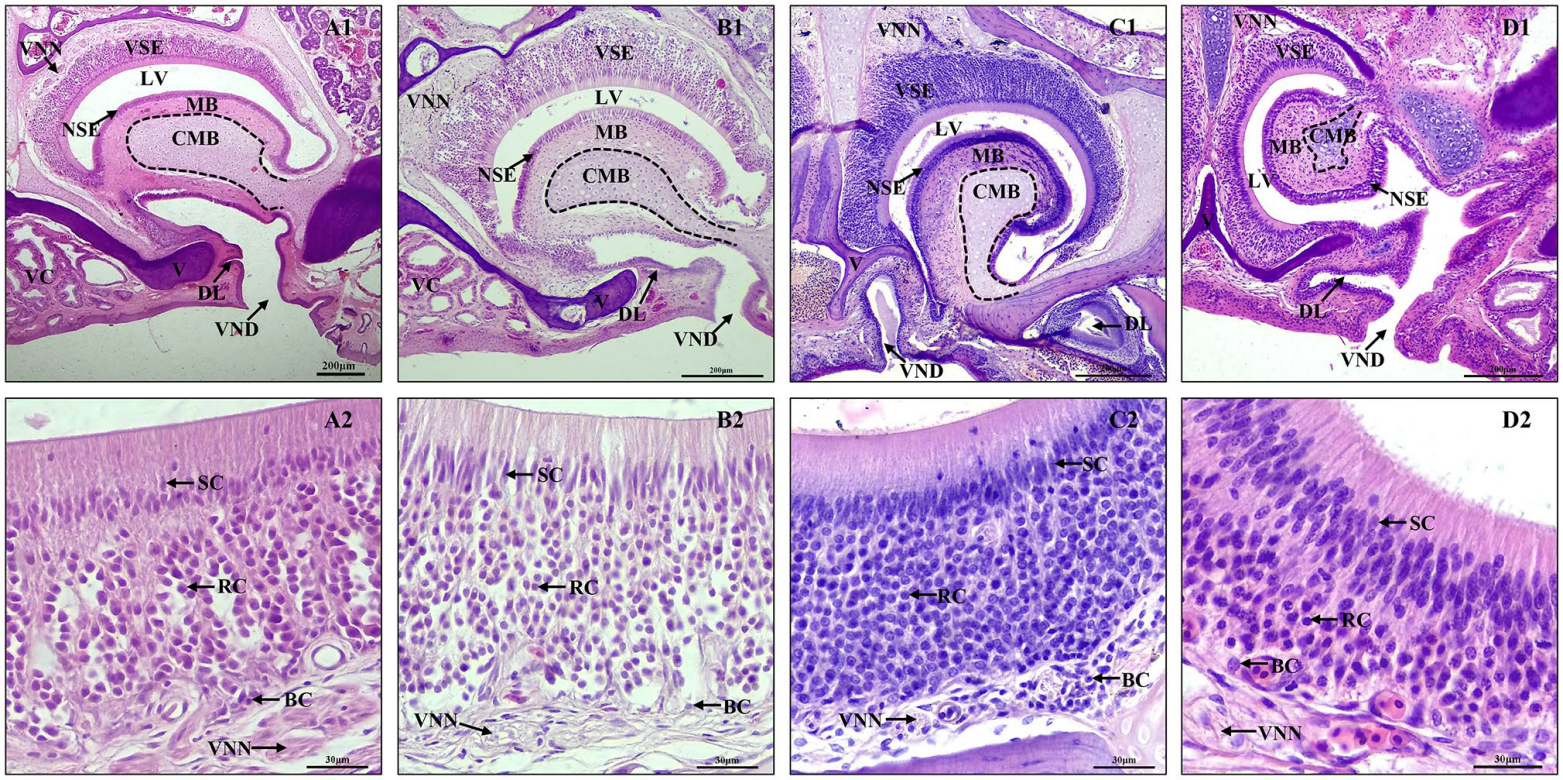
Transverse sections of the VNO in four species of lizards. (A1, A2) *T. roborowskii* (active foraging); (B1, B2) *T. dadunensis* (sit‐and‐wait foraging); (C1, C2) *E. roborowskii* (active foraging); (D1, D2) *P. axillaris* (sit‐and‐waitl foraging). BC, basal cell; CMB, cartilage of the mushroom body; LD, lacrimal duct; LV, lumen of the VNO; MB, mushroom body; NSE, non‐sensory epithelium; RC, receptor cell; SC, supporting cell; V, vomer; VC, vomerine cushion; VND, VNO duct; VNN, vomeronasal nerve; VSE, vomeronasal sensory epithelium.

The VNS of the four species of lizards consisted mainly of the lumen of the VNO, VSE, mushroom body, and vomeronasal nerve; no glandular structures were found (Figure 8). Three‐dimensional (3D) reconstructions of the lacrimal ducts and vomeronasal organ (VNO) in four lizard species demonstrate that the Harderian gland connects to the VNO through the lacrimal ducts (Figure 2A3,B3,C3,D3). The mushroom body of the four species of lizards was covered with ciliated non‐sensory epithelium (Figure 8). The position of the mushroom body within the lumen of the organ varies considerably (Figures 6 and 8). The mushroom body of *T. roborowskii* , *T. dadunensis*, and *E. roborowskii* takes origin from the floor of the lumen of the VNO (Figure 6). The VNO duct and the ventral channel are located on the opposite sides of the mushroom body (Figure 6A1,B1,C1). But the mushroom body of *P. axillaris* is somewhat differently oriented than in the three species of lizards. It appears tipped medially, the mushroom body originates from dorsolaterally of the lumen of the VNO, possibly in relation to flattening of the snout (Figure 6D1,D2). Its VSE is hence best developed medially, becoming reduced laterally.

**FIGURE 9:**
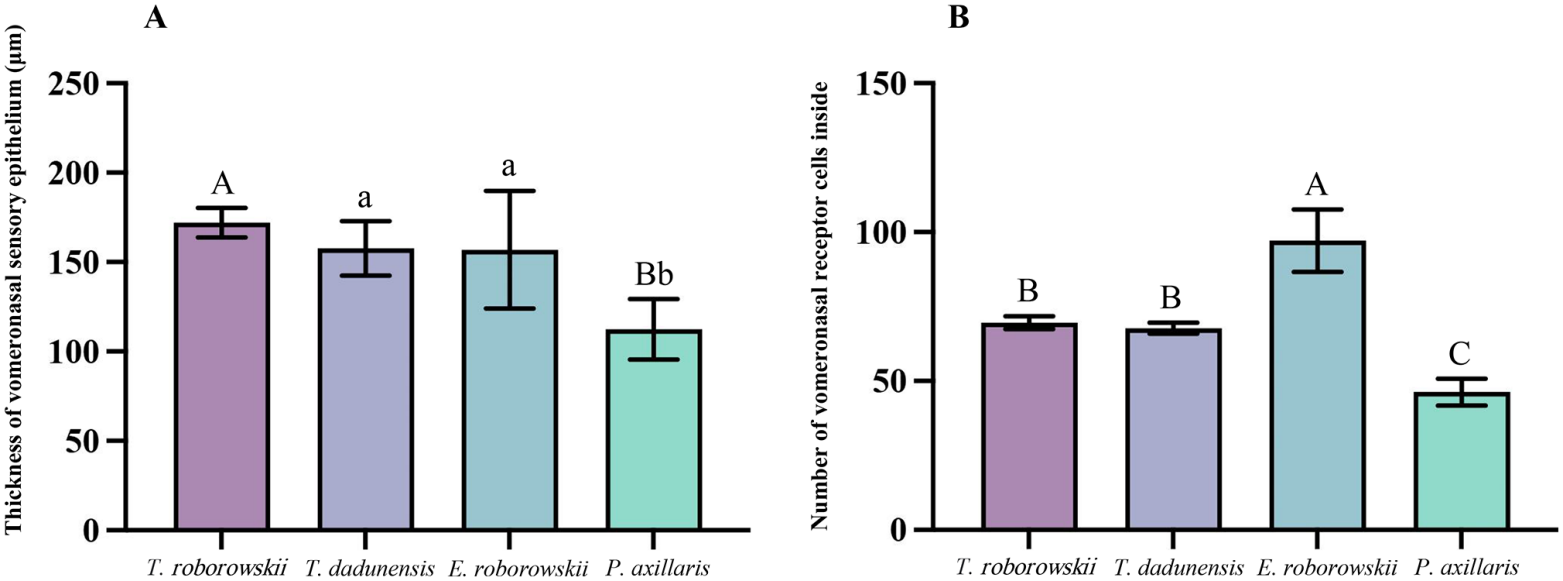
Histological parameter of the vomeronasal epithelium in four species of lizards. (A) Thickness of the vomeronasal sensory epithelium; (B) number of vomeronasal receptor cells within a 60 × 60 μm^2^ area. Groups not sharing the same lowercase letter in each cluster are significantly different (*p* < 0.05). Groups not sharing the same capital letter in each cluster are highly significantly different (*p* < 0.01). *T. roborowskii* (active foraging); *T. dadunensis* (sit‐and‐wait foraging); *E. roborowskii* (active foraging); *P. axillaris* (sit‐and‐wait foraging).

There was a significant difference in the thickness of the VSE of the four species of lizards (*p* = 0.015) (Table 2). The thickness of the VSE of *P. axillaris* was highly significantly smaller than that of *T. roborowskii* (*p* = 0.004), significantly smaller than that of *T. dadunensis* (*p* = 0.016), and significantly smaller than that of *E. roborowskii* (*p* = 0.017) (Figure 9A). Highly significant differences in the number of vomeronasal receptor cells of the four lizard species within a 60 × 60 μm^2^ unit area at 400X magnification (*p* < 0.001) (Table 2). The number of vomeronasal receptor cells per unit area of *E. roborowskii* was highly significantly more than that of *T. roborowskii* (*p* < 0.001), highly significantly more than that of *T. dadunensis* (*p* < 0.001), and highly significantly more than that of *P. axillaris* (*p* < 0.001). The number of vomeronasal receptor cells per unit area of *P. axillaris* was highly significantly smaller than that of *T. roborowskii* (*p* < 0.001), and highly significantly smaller than that of *T. dadunensis* (*p* = 0.001) (Figure 9B).

## 4 Discussion

Sympatric lizards frequently exhibit distinct foraging modes that directly influence their behavioral patterns, morphological traits, and ecological performance (Miles et al. 2007; Donihue 2016). Notably, such transitions between foraging modes are hypothesized to induce adaptive morphological variations in the olfactory system among lizard species, as demonstrated by comparative analyses of sensory organ structures (Baeckens, Van Damme, et al. 2017). The present study is the first such investigation to use DiceCT and histological sections to compare the fine structure of the olfactory system in sympatric lizards with different foraging modes. Study results support the hypothesis that foraging modes drive morphological variation in the olfactory systems among lizards (Baeckens, Van Damme, et al. 2017).

### 4.1 Morphological and Histological Differences of NOS

The morphology of the vestibulum exhibits great variation in adult squamate reptiles (Stebbins 1948). The vestibulum of adult squamate reptiles is putatively responsible for the removal of dust from the respiratory tract (Pratt 1948). This function is improved by the epithelium hyperplasia of the vestibulum, spongy sinusoidal tissue, and the lateral nasal gland producing mucoid secretion (Pratt 1948; Stebbins 1948). A deserticolous existence is associated with marked elongation of this vestibulum (Stebbins 1948). Becoming exceptionally well marked in deserticolous lizards (Stebbins 1948). It is worth mentioning that the degree of development of the lateral nasal gland is thought to be related to the functional importance of the vestibulum (Stebbins 1948). All four lizard species from Turpan in this study exhibited adaptations to extreme desert environments in both the vestibulum and lateral nasal glands (Figure 2). For example, the curved vestibulum and lateral nasal glands of *E. roborowskii* may suggest adaptations to hot desert environments (Figure 2C1,C2). Interestingly, the lateral nasal glands appeared less developed in the diurnal *P. axillaris* compared to *E. roborowskii*; however, *P. axillaris* may adapt to hot desert conditions through alternative mechanisms, including potentially modifiable nasal valve function (Zhao et al. 1999) and vestibulum curvature (Figure 2D1,D2).

The NOS primarily senses small, highly volatile molecules that readily diffuse into the air and can travel long distances (Filoramo and Schwenk 2009). These airborne volatile molecules enter the nasal cavity during respiration and are detected by olfactory nerves after dissolution in nasal fluid. This fluid is secreted primarily by Bowman's glands and supporting cells (Kratzing 1975). The distribution area of the OE shows a direct correlation with the nasal cavity's anatomical configuration (Rehorek et al. 2000a). Because the OE is restricted to the dorsal surface of the Stammteil (main nasal cavity), any increase in OE surface area can only occur through expansion of the sensory epithelium (Sapoznikov et al. 2016). This can be achieved through either enlarging the Stammteil or introducing external convexities/internal depressions to its structure (Rehorek et al. 2000a). In squamates, the lateral nasal concha (a convex structure forming the lateral nasal wall) effectively expands the dorsal surface area covered by OE (Rehorek et al. 2000a). The OE is extensively distributed over the dorsal surface of the lateral nasal concha. A large and well‐developed lateral nasal concha can significantly increase the surface area covered by the OE (Rehorek et al. 2000a; Yohe et al. 2018). Therefore, variations in the size of the nasal concha (and consequently the distribution of the OE) may correlate with olfactory acuity. DiceCT and 3D reconstruction revealed that *T. roborowskii* , *T. dadunensis*, and *E. roborowskii* possessed well‐developed lateral nasal conchae, whereas *P. axillaris* exhibited degenerated conchae (Figures 2 and 3). The enlarged lateral nasal conchae of the active foraging *E. roborowskii* facilitated expansion of the Stammteil (nasal main chamber), a feature markedly reduced in the sit‐and‐wait foraging *P. axillaris* (Figures 2 and 3). Furthermore, *E. roborowskii* exhibited a statistically significant increase in olfactory receptor cell density per unit area compared with *P. axillaris* (*p* = 0.000, Figure 5B). The nasal cavity of *T. roborowskii* and *T. dadunensis* is morphologically similar, both exhibiting enlarged nasal cavities and dilated lateral nasal conchae (Figure 2). Their nasal cavity structures closely resemble those of the gecko species *Lepidodactylus lugubris* and *Eublepharis macularius* (Kaczmarek et al. 2021). The unique combination of large nasal concha (greater surface area of the OE) and a small number of granules in the supporting cells supports the idea that geckos are olfactory specialists (Dial and Schwenk 1996; Schwenk 1993a). Our results revealed that although both *T. roborowskii* and *T. dadunensis* possess well‐developed lateral nasal turbinates and abundant OE, the seasonal frugivorous *T. roborowskii* —which employs an active foraging strategy to locate fixed and concealed *C. spinosa* fruits—exhibited significantly greater OE thickness (*p* = 0.001) and a higher density of olfactory sensory cells per unit area (*p* = 0.001) compared to the sit‐and‐wait foraging *T. dadunensis* (Figure 9). This suggests that active foraging *T. roborowskii* exhibited significantly greater capability to detect small, highly volatile molecules compared to sit‐and‐wait foraging *P. axillaris* .

### 4.2 Morphological and Histological Differences of VNS

VNS consists of the lumen of the VNO, VSE, vomeronasal nerve, and mushroom body (Baeckens, Van Damme, et al. 2017). In snakes and lizards, VNS completely lost its anatomical connection to NOS; VNO is separated from the nasal cavity and communicates with the oral cavity through the VNO duct (Døving and Trotier 1998). In four species of lizards, VNO is connected to the oral cavity through the VNO duct, and VNO has no anatomical connection to the nasal cavity (Figure 6). The lumen of the VNO of squamate reptiles is a fluid‐filled space (Halpern 1987). But no gland structures were found in any VNS of the four species of lizards (Figure 8), suggesting an external source of fluid in the lumen of the VNO (Rehorek et al. 2000b). Most of the fluid in the lumen of the VNO of squamate reptiles is produced in the Harderian gland (Rehorek et al. 2000b, 2003). The Harderian gland is a large secretory structure located near the orbit that specializes in secreting proteins and mucus into the lacrimal duct (Rehorek et al. 2000a, 2000b). A follow‐up study confirmed the path of Harderian gland secretions through the lacrimal duct into the lumen of the VNO (Bentz 2019). The absence of the vomeronasal gland is compensated by the entry of the Harderian gland secretions into the lumen of the VNO through the lacrimal duct (Saito et al. 2010). In our study, the 3D reconstruction results of the VNO and lacrimal duct also confirmed the connection between the VNO and the Harderian gland (Figure 2). In addition, the protein components secreted by the Harderian gland are thought to be important for the function of the VNS, which helps to detect nonpolar, water‐insoluble chemical cues (Mason and Halpern 2011).

Although the VNO is located ventrally to the nasal cavity in all studied species, the position of the mushroom body within the lumen of the VNO varies considerably (Døving and Trotier 1998). The mushroom body of *T. roborowskii* , *T. dadunensis*, and *E. roborowskii* takes origin from the floor of the lumen of the VNO (Figure 6). The VNO duct and the ventral channel are located on the opposite sides of the mushroom body (Figure 6A1,B1,C1). This is consistent with *Takydromus sexlineatus* (Baeckens, Herrel, et al. 2017), *Eremias multiocellata* (Han et al. 2024), *Scincella tsinlingensis* (Yang et al. 2020), *Takydromus tachydromoides* (Saito et al. 2010), *Lepidodactylus lugubris* , and *Eublepharis macularius* (Kaczmarek et al. 2021). Differently, the mushroom body of *P. axillaris* appears tipped medially; the mushroom body originates from dorsolaterally in the lumen of the VNO, possibly in relation to flattening of the snout (Figure 6D1,D2). Its VSE is hence best developed medially, becoming reduced laterally. Its morphological structure is similar to that of *P. frontalis* and *P. przewalskii* (Han et al. 2024). The position of the mushroom body within the lumen of the VNO may reflect the receptivity of different lizards VNO to chemical information.

Chemical signaling molecules must first be transported through a pair of the VNO ducts into the lumen of the VNO before they can be recognized by the VSE (Halpern 1987). The location of the opening of the VNO duct varies by taxon. The VNO duct opening is located just above the front of the tongue; chemical signaling molecules are passed through the tongue‐flick. The morphology of the VNO and tongue varies considerably among different squamate reptile taxa; this reflects the differential reliance of squamate reptiles on the VNS (Cooper 1997a, 1997b). Taxa with elongated, forked tongues generally have thicker VSE and more vomeronasal receptor cells, which can effectively enhance the sampling and processing of chemical odors (Baeckens, Herrel, et al. 2017). The tongue of active foraging *E. roborowskii* is elongated and has a distinctly forked tip; the tongue of sit‐and‐wait foraging *P. axillaris* is wide, short, and unforked. The thickness of the VSE (*p* = 0.017) and the number of vomeronasal receptor cells per unit area (*p* = 0.000) were significantly greater in *E. roborowskii* than in *P. axillaris* (Figure 9). In addition, *E. roborowskii* with long, forked tongues was found to have frequent tongue‐flicks during foraging. The two nocturnal *T. roborowskii* and *T. dadunensis* have similar tongue morphology; the tongue is long and wide, and the tip of the tongue is also bifurcated. Although significant differences exist in the foraging modes of *T. roborowskii* and *T. dadunensis*, there were no significant differences in the thickness of the VSE or the number of vomeronasal receptor cells per unit area (*p* > 0.05; Figure 9).

Diurnal *Naultinus manukanus* use chemical cues to identify and locate fruits (Hoare et al. 2007). However, changes in this behavior were not accompanied by changes in tongue‐flick rate. Therefore, fruit location recognition may be mainly caused by NOS cues rather than VNS cues (Cooper and Pérez‐Mellado 2001; Wotton 2002). *Podarcis lilfordi* are able to locate odorous plant food from a distance and differentiate between food and non‐food by tongue‐flick to contact the substrate (Cooper and Pérez‐Mellado 2001). Therefore, we hypothesized that seasonal frugivorous *T. roborowskii* mainly senses and locates *C. spinosa* fruits through the NOS, since most plant fruits odor components are small molecule of high volatility (Nevo and Valenta 2018). The VNS may play an important role in identifying fruit quality and ripeness from close range. The results of the swab experiments also revealed significant differences in the rate of tongue‐flick by *T. roborowskii* on the leachate of *C. spinosa* fruits of different maturity levels (He et al. 2018a).

### 4.3 Phylogenetic Constraints and Evolutionary Lability

This study examines four sympatric lizard species from the same habitat to reduce the impact of varying environmental factors on their olfactory systems (Donihue 2016). Despite belonging to distinct families (Lacertidae, Agamidae, Gekkonidae, Sphaerodactylidae), the lizard's olfactory systems exhibited convergence linked to foraging ecology rather than strict phylogenetic heritage. For instance, the active‐foraging lizards *E. roborowskii* (Lacertidae) and *T. roborowskii* (Sphaerodactylidae) both evolved robust VNS structures despite their divergent lineages, highlighting adaptive lability in response to similar ecological pressures (Donihue 2016). Conversely, *P. axillaris* (Agamidae) showed the typical traits (simplified nasal cavity, reduced VSE) associated with the sit‐and‐wait foraging mode. Similar to the findings from studies on *Phrynocephalus frontalis*, *Phrynocephalus przewalskii* (Han et al. 2024), *Anolis sagrei* (Kaczmarek et al. 2020), and *Iguana iguana* (Sapoznikov et al. 2016). Our findings are consistent with the characteristic visual predation behavior of Agamidae (Baeckens, Van Damme, et al. 2017). However, exceptions within families—such as gekkonids with intermediate morphologies—emphasize that foraging mode can override phylogenetic inertia, particularly in taxa occupying variable niches (Cooper and Habegger 2000; Wehsener and Noss 2022). This lability may suggest that sensory evolution in squamates could be shaped by a dynamic interplay between lineage‐specific constraints and ecological opportunity. To explore this further, future work should expand comparative analyses to encompass a broader diversity of squamate taxa—including understudied lineages and representatives from varied ecological contexts—would help refine our understanding of this potential interplay. Such studies could explicitly test whether the observed patterns of lability align with the proposed balance between lineage constraints and ecological opportunity, thereby clarifying the generalizability of these evolutionary dynamics across squamates.

## 5 Conclusion

Our results support the hypothesis that foraging mode drives morphological variation in the olfactory systems of lizards. The active foraging *E. roborowskii* exhibited better developed NOS and VNS compared to the sit‐and‐wait foraging *P. axillaris* . The seasonal frugivorous *T. roborowskii* , which adopts an active foraging strategy to locate fruits, showed a more developed NOS than the sit‐and‐wait foraging *T. dadunensis*. These results provide morphological and histological evidence for differences in NOS and VNS among lizards with different foraging modes; further studies are needed to investigate their functional implications.

## Author Contributions

**Lin Leng:** conceptualization (equal), data curation (equal), formal analysis (equal), investigation (equal), methodology (equal), project administration (equal), software (equal), supervision (equal), validation (equal), visualization (equal), writing – original draft (equal), writing – review and editing (equal). **Lei Shi:** conceptualization (equal), data curation (equal), formal analysis (equal), funding acquisition (lead), methodology (equal), project administration (equal), resources (lead), supervision (equal), validation (equal), writing – original draft (equal), writing – review and editing (equal).

## Ethics Statement

The authors declare that this article is reported in accordance with the ARRIVE guidelines 2.0. The study has been conducted under the approval of a permit issued by the Ethics in Animal Experimentation Committee of Xinjiang Agricultural University (Approval No. 2023013).

## Conflicts of Interest

The authors declare no conflicts of interest.

## Supporting information


**Table S1:** List of abbreviations.
**Table S2:** Results of power analysis.

## Data Availability

The data for this study are available on Figshare (https://doi.org/10.6084/m9.figshare.27087064.v1).
