# Supplementary material for: How Foraging Mode Sculpts Sensory Systems: Morphological Evidence From DiceCT and Histology in Sympatric Lizards
Source: Ecol Evol. 2025 Aug 21;15(8):e72042. doi: 10.1002/ece3.72042 (PMC12371125; doi:10.1002/ece3.72042)
Supplement: Supplementary file 1 — Table S1: List of abbreviations. Table S2: Results of power analysis. [file ECE3-15-e72042-s001.docx]

**How foraging mode sculpts sensory systems: morphological evidence from DiceCT and histology in sympatric lizards**

Lin Leng, Lei Shi*

Xinjiang Key Laboratory for Ecological Adaptation and Evolution of Extreme Environment Organism, College of Life Sciences, Xinjiang Agricultural University, Urumqi 830052 China.

*Corresponding author: Lei Shi

1. mail: [leis@xjau.edu.cn](mailto:leis@xjau.edu.cn)

**Supplementary materials**

**Supplementary Table 1. List of abbreviations**

| **[Abbreviation](#abbreviation)** | **Full name** |
| --- | --- |
| BC | basal cell |
| BG | Bowman’s glands |
| CH | choana |
| CI | cilia |
| ECS | extra-conchal space |
| EN | external naris |
| LNC | lateral nasal concha |
| LNG | lateral nasal gland |
| LV | lumen of the vomeronasal organ |
| MB | mushroom body |
| NSE | non-sensory epithelium |
| NOS | nasal olfactory system |
| NS | nasal septum |
| OE | olfactory epithelium |
| ON | olfactory nerve |
| RC | receptor cell |
| SC | supporting cell |
| STT | Stammteil |
| T | tongue |
| VC | vomerine cushion |
| VES | vestibulum |
| VND | vomeronasal duct |
| VNN | vomeronasal nerve |
| VNS | vomeronasal system |
| VSE | vomeronasal sensory epithelium |

**Supplementary Table 2. Results of power analysis**

|  | Thickness of the olfactory epithelium (**μ**m) | Number of olfactory receptor cells within a 60 × 60 **μ**m² area | Thickness of the vomeronasal sensory epithelium(**μ**m) | Number of vomeronasal receptor cells within a 60 × 60 **μ**m² area |
| --- | --- | --- | --- | --- |
| SD | 9.18 | 8.40 | 20.39 | 6.60 |
| Effect size f | 1.355 | 2.426 | 1.162 | 2.800 |
| Actual power | 0.871 | 0.999 | 0.955 | 0.999 |
